# Long-Term Assessment of Wild Boar Harvesting and Cattle Removal for Bovine Tuberculosis Control in Free Ranging Populations

**DOI:** 10.1371/journal.pone.0088824

**Published:** 2014-02-18

**Authors:** Gregorio Mentaberre, Beatriz Romero, Lucía de Juan, Nora Navarro-González, Roser Velarde, Ana Mateos, Ignasi Marco, Xavier Olivé-Boix, Lucas Domínguez, Santiago Lavín, Emmanuel Serrano

**Affiliations:** 1 Servei d′Ecopatologia de Fauna Salvatge (SEFaS), Departament de Medicina i Cirurgia Animal, Facultat de Veterinària, Universitat Autònoma de Barcelona, Bellaterra, Barcelona, Spain; 2 VISAVET Health Surveillance Centre. Universidad Complutense, Madrid, Spain; 3 Departamento de Sanidad Animal. Facultad de Veterinaria, Universidad Complutense, Madrid, Spain; 4 Estadística i Investigació Operativa, Departament de Matemàtica. Universitat de Lleida, Lleida, Spain; 5 Reserva Nacional de Caça dels Ports de Tortosa i Beseit, Roquetes, Tarragona, Spain; National Institute for Agriculture and Veterinary Research, IP (INIAV, I.P.), Portugal

## Abstract

Wild boar is a recognized reservoir of bovine tuberculosis (TB) in the Mediterranean ecosystems, but information is scarce outside of hotspots in southern Spain. We describe the first high-prevalence focus of TB in a non-managed wild boar population in northern Spain and the result of eight years of TB management. Measures implemented for disease control included the control of the local wild boar population through culling and stamping out of a sympatric infected cattle herd. Post-mortem inspection for detection of tuberculosis-like lesions as well as cultures from selected head and cervical lymph nodes was done in 745 wild boar, 355 Iberian ibexes and five cattle between 2004 and 2012. The seasonal prevalence of TB reached 70% amongst adult wild boar and ten different spoligotypes and 13 MIRU-VNTR profiles were detected, although more than half of the isolates were included in the same clonal complex. Only 11% of infected boars had generalized lesions. None of the ibexes were affected, supporting their irrelevance in the epidemiology of TB. An infected cattle herd grazed the zone where 168 of the 197 infected boars were harvested. Cattle removal and wild boar culling together contributed to a decrease in TB prevalence. The need for holistic, sustained over time, intensive and adapted TB control strategies taking into account the multi-host nature of the disease is highlighted. The potential risk for tuberculosis emergence in wildlife scenarios where the risk is assumed to be low should be addressed.

## Introduction

### Epidemiology

The ever-growing impact of wildlife reservoirs in the epidemiology of bovine tuberculosis (TB) has become clear in the recent decades worldwide [1]. Hence, although the effectiveness of TB eradication programs in cattle has been considerable [2], success also depends on the absence or control of wildlife reservoirs [3]–[5]. Under natural conditions, the multi-host nature of this disease would render ineffective any control strategy that overlooks the ecology, susceptibility, behaviour and abundance of the whole host community [4]–[6]. As a result, understanding the role of each host species in the maintenance and transmission of the disease is essential to designing any measures for TB control [1], [6].

In the last decade, a large number of species have been identified as spill-over, maintenance and/or reservoir hosts in the wild [1]. Wild boar (*Sus scrofa*) and, to a lesser extent, red deer (*Cervus elaphus*) are considered maintenance hosts of TB in the Iberian Peninsula and often suggested as reservoirs for livestock [7]–[9]. The link between wild and domestic ungulates in the epidemiology of TB has been confirmed in southern Spain through genotype mapping [10], [11]. This finding along with the persistence of high TB prevalence in wild boar populations isolated from livestock for decades and lesion pattern characteristics indicating infection and excretion routes have been key factors for recognizing its role as a true reservoir in the Mediterranean ecosystems [8]. This situation is especially concerning for EU animal health policies given the huge increase in wild boar populations [12]. The problem is magnified in southern Spain mainly by the existence of estates where extensive livestock coexist with managed wild ungulate populations with up to 90 individuals/km^2^ aimed at commercial hunting [12], [13]. In contrast to this, game management of wildlife is anecdotal in the northern part of the country and generally in Europe [7], [9], [14].

### Management

In line with compulsory tests and slaughter campaigns implemented in livestock, enormous efforts are underway for TB control in wildlife [6], [15]–[18]. Different strategies have been adopted for this purpose, with culling of the reservoir the most common in the case of game, feral or pest species. In some cases, wildlife culling may be socially unacceptable [19], not sufficiently effective [16] or even counter-productive [20] in such a way that additional or alternative measures for TB control are necessary [15], [21]. However, since game ungulates are common TB reservoirs in the wild, this strategy may be a suitable option that can be applied as major indiscriminate depopulation exercises or more restricted culling protocols aimed at reducing reservoir density below the theoretical persistence threshold [15], [22] or using “capture-test-and-slaughter” protocols where only positive animals are culled [23] in order to appease social and economic concerns [19]. Regarding wild boar, little and contradictory information on the effectiveness of intensive culling for TB control has been recently published. Under similar field (fencing and high densities of ungulates) and treatment conditions, Boadella and cols. succeeded in decreasing TB prevalence by 21–48% after reducing wild boar abundance to half [17], while Garcia-Jimenez and cols. failed to do so [18].

Herein we report the output of eight years of TB monitoring in wild boar, cattle and Iberian ibex (*Capra pyrenaica hispanica*) in a different scenario to that previously described for the Mediterranean ecosystems. Specifically, this is the first high prevalence focus of tuberculosis in wildlife in northern Spain, occurring in a free-ranging (non-fenced), non-intensively managed wild boar population sharing habitat with cattle and ibexes in a national game reserve. In addition, this area lacks other known wild reservoirs of TB in the Iberian Peninsula, red deer (*Cervus elaphus*) and fallow deer (*Dama dama*). This focus of tuberculosis was first detected in 2004 in hunter-harvested wild boar thanks to active disease surveillance in wildlife. We also show the outcome of the combination of two different disease management strategies consisting of intensive culling of wild boar populations and removal of a sympatric infected cattle herd. This is the first attempt to address control of TB in a wild boar population through eradication of sympatric infected cattle (most probably, the original source of infection for wild boar in our study area) in the Mediterranean context. Our main objectives were: (i) to characterize the epidemiology of TB amongst sympatric wild boar, cattle and Iberian ibexes through both the study of macroscopic lesion patterns in wild boar and the genotyping of *Mycobacterium tuberculosis* complex isolates obtained; (ii) to describe spatial and temporal TB patterns in this scenario; (iii) to evaluate whether the implemented disease management strategies succeeded in the control of TB; (iv) and to explore the effects of the intensive culling on the wild boar population structure to better understand the effect of this measure on TB evolution.

## Materials and Methods

### Ethics statement

No approval was needed from any Ethics committee since the animals used in the present study were not sacrificed for research purposes. The harvested wild boar and ibexes included in the present study have been legally hunted (shot) or box-trapped in their own habitat by authorized gamekeepers and hunters within the framework of an annual hunting plan approved by the Departament d′Agricultura, Ramaderia, Pesca, Alimentació i Medi Natural - Generalitat de Catalunya (DARPAMN -the Regional authority in charge of livestock and wildlife management-). Box-trapping and euthanasia of wild boar was promoted and approved by the DARPAMN as an exceptional measure for the control of bovine tuberculosis in the affected area. The bovine tuberculosis positive cows were slaughtered (shock and bleed) in an authorised abattoir according to the guidelines of the Council Directive 64/432/EEC and subsequent modifications on animal health problems affecting intra-Community trade in bovine animals and swine. Hence, no animals were harvested in order to perform this study, but we took advantage of the harvested animals for this aim. Standard protocols of anaesthesia and euthanasia were used to minimize stress and suffering of the box-trapped wild boar and carried out by veterinarians.

### Study area

This focus of tuberculosis is located in the National Game Reserve “Ports de Tortosa i Beseit” (NGRPTB) in north-eastern Spain (40°48′ 28′′N, 0 19′ 17′′ E) and within the Iberian bio-region 5, as described in [24]. It is a limestone mountain massif of about 28,000 ha that shows a high level of orographic complexity, which results in a rugged and abrupt terrain formed by numerous canyons, ravines and steep slopes. About 28% of the surface is above 1000 m.a.s.l., with the highest peak being Mont Caro (1442 m) and the lower heights around 300 m.a.s.l. The mean annual temperature in the reserve is 13.7°C (min = 1.6°C in December – February, max = 30°C in July – August), while the mean annual accumulated rainfall is 697 mm (min = 536 in 2009, max = 889 in 2011) [25]. The vegetal stratum is characterized by a typical Mediterranean forest dominated by *Quercus ilex* and *Pinus halepensis* with dense scrublands of *Quercus coccifera*, *Pistacea lentiscus* and *Chamaerops humilis*, among others. Patches of non-irrigated crops are also common in the study area, mainly those with olive trees (*Olea europaea*), European carobs (*Ceratonia siliqua*) and almond trees (*Prunus amygdalus*). The average density of Iberian ibexes is 11.1 individuals/km^2^ (Personal communication; Distance sampling® estimate by the NGRPTB managers) and of wild boar is 3 individuals/km^2^ (estimate based on hunting bags for the whole NGRPTB), the only wild ungulates that share grazing areas with cattle (cross-breed of Spanish fighting bull) year-round. The farming conditions of cattle in the study area are free-ranging (extensive farming) with supplemental feeding in the dry season (summer).

**Figure 1:**
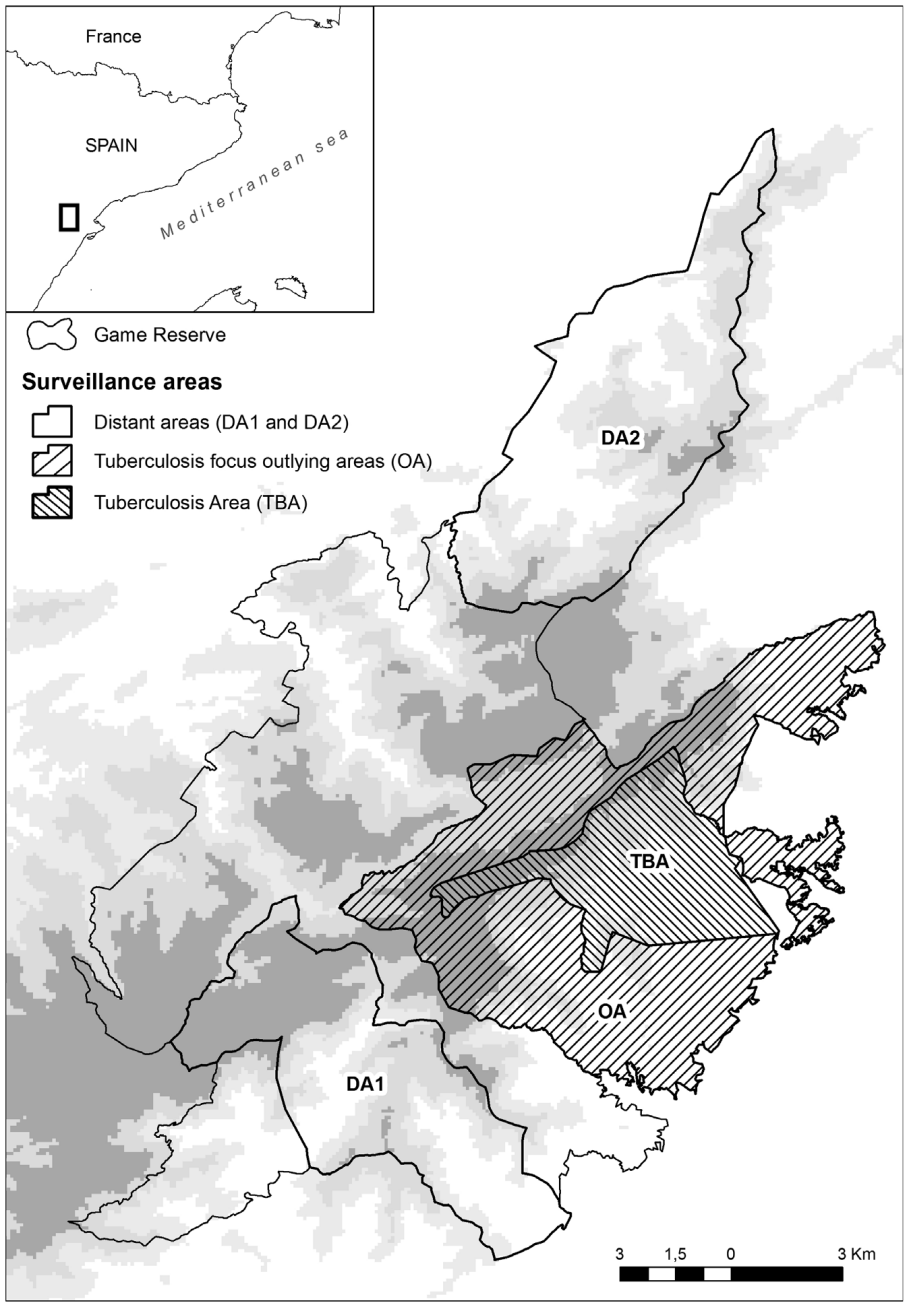
Study area. The study area is located in the National Game Reserve “Ports de Tortosa i Beseit”, Catalonia region, north-eastern Spain. Three different areas were defined according to preliminary apparent TB distribution: the main tuberculosis area (TBA), outlying areas to TBA (OA) and two distant areas (DA1 and DA2) to TBA.

Ravines and other natural barriers may play an important role driving transmission of infectious diseases [26]. Hence, based on the local orography and the preliminary observations on the TB distribution, we defined three different zones within the study area (Figure 1): TBA (defined as “tuberculosis area”), an area of 2,150 ha where the first cases of TB in wild boar were detected and the disease management has been carried out; OA ("outlying areas”) covers 6,380 ha of the surrounding areas that could be potentially affected by the spread of TB from TBA; and DA (“distant areas to TBA”; 8,810 ha.), consisting of two different zones [DA1 and DA2; 3,920 and 4,890 ha., respectively], where TB-positive wild boar have also been detected and TB surveillance has been maintained during the whole study period. No disease management actions have been carried out in either OA or DA.

### Study period

Tuberculosis-like lesions (TBLL) were first detected in the TBA in December 2004, while performing field necropsies of hunter-harvested wild boar, and were later confirmed by culture and isolation of *Mycobacterium bovis.* Since then, wild boar have been harvested by hunting and box-trapping captures year-round, with a peak harvest time in autumn-winter (September – March), coinciding with the regular game season. For this reason, we divided the TB study period (2004–2012) into eight harvesting periods covering July to June of the following year (from 2004–05 to 2011–12). These periods were conceived to include the regular game seasons in the middle of every harvesting period. To analyse the accumulated effect of culling, data regarding hunting pressure and wild boar abundance was used from 2001 onward. Thus, the first harvesting period (2001-2002) corresponds to number 1, and so on.

### Animal inspection and sampling

During the study period, 745 wild boar (436 in TBA, 209 in OA and 100 in DA), 355 Iberian ibexes (throughout NGRPTB) and five cows (from the TB affected bullfighting herd in TBA and with a positive result for the tuberculin skin test) were inspected for TBLL. Wild boar were either hunter-harvested during the regular game seasons (in the whole NGRPTB; n = 591) or box-trapped and later euthanatized year-round (only in TBA and in harvesting periods number 6 to 11; n = 154). Complete post-mortem examination and, thus, determination of the distribution of the lesions was possible in 115 (39 box-trapped and 76 hunter-harvested) tuberculous wild boar. In agreement with the authorities responsible for the management of game in the NGRPTB, their game rangers were trained to identify the normal aspect of organs and responsible for collecting apparently abnormal organs and all the mandibular and retropharyngeal lymph nodes of Iberian ibexes hunter-harvested during the regular game season for this species (two annual periods in spring -March to June- and autumn -September to December-, respectively). Finally, the five cows were inspected and sampled in the slaughterhouse facilities following the established official channels. Once inspected for TBLL, selected samples were either refrigerated in a cold box (4°C) and immediately dispatched to the laboratory for detection of Mycobacteria within the first 48–72 hours (wild boar and cows) or stored at −20°C until processing (ibex).

Sex and age of the animals was recorded. Wild boar were aged by tooth replacement and by dental attrition [27] but ultimately assigned to four age classes for minimizing determination errors: piglets (0–6 months; n = 115), juveniles (6–12 months; n = 152), yearlings (13–24 months; n = 95) and adults (over 24 months; n = 383). The age of ibexes was determined in years by counting horn segments [28].

### Bacteriology

Selected samples (mostly head and cervical lymph nodes and including TBLL if present) from every animal were subjected to bacteriological culture regardless of TBLL presence or not. Samples from each animal were pooled, homogenized with sterile distilled water and decontaminated with 0.35% hexadecylpyridinium chloride for 30 minutes [29], centrifuged at 1300 g for 30 min and cultured onto Coletsos and 0.2% (w/v) pyruvate-enriched Löwenstein-Jensen media (Biomedics, Madrid, Spain) at 37°C. Isolates were heat-treated and identified by PCR amplification of Mycobacterium genus-specific 16 S rRNA fragment and the MPB70 sequence for the *M. tuberculosis* complex (MTBC) isolates [30].

### Fingerprinting

The DVR-spacer oligonucleotide typing (DVR-spoligotyping) method was later performed as previously described [31] to identify the mycobacterium species of the MTBC and to characterize the isolates. In addition, data of spoligotypes detected in cattle grazing in our study area were obtained from the Spanish Database of Animal Mycobacteriosis (mycoDB) [32] and the authorities in charge of the compulsory test and slaughter campaigns. Mycobacterial Interspersed Repetitive Units - Variable Number Tandem Repeats (MIRU-VNTR) typing was also performed using nine VNTR markers (ETR-A, ETR-B, ETR-D, ETR-E, MIRU26, QUB11a, QUB11b, QUB26 and QUB3232) [33].

For further analysis, TB infection status was based on both gross tubercle-like lesions (TBLL) and/or microbiological culture, in order to alleviate disease underestimation [34].

### Increase of wild boar harvesting

Once bovine tuberculosis was detected (in 2004), it was decided to increase wild boar harvesting in the affected area with the aim of disease control. Specifically, the strategy was based on the authorization of additional hunting battues and the implementation of a box-trapping system in the TBA. This consists of six box-traps permanently baited with acorns and activated monthly. Box-trapped boars were anaesthetized with a combination of xylazine (3 mg/kg IM; Xilagesic®, Calier Laboratories), zolazepam and tiletamine (3 mg/kg IM each; Zoletil®, Virbac Laboratories) delivered by blowpipe and then euthanized with T-61 euthanasia solution (0.1 mL/kg IV; T-61®, Intervet Laboratories).

### Cattle removal

Bovine tuberculosis is subjected to compulsory tests and slaughter campaigns in cattle (EC No 64/432 and the Spanish transposition R.D. 2611/1996) with the higher infection rates occurring in beef and bullfighting cattle, mainly in south-central Spain [35]. Due to repeated TB positive cases amongst tested cattle (bullfighting) in the TBA area, compulsory removal and slaughter (“stamping out”) of the entire herd population was officially decreed by May 2008 and, after a 13-month period, a new TB-free herd was reintroduced into the TBA.

### Statistical analysis

Different statistical analyses were performed depending on the objectives of the study. To assess the spatial pattern of TB in the game reserve, the differences in TB prevalence among TBA, OA and DA zones were analysed by a Fisher's exact test [36]. To assess the effects of disease management on TB control, we first checked whether the yearly increases in harvesting pressure, including both hunting and box-trapping sessions, resulted in an increase in harvested boars. This was checked by a linear regression between the number of harvesting sessions and the number of wild boar harvested. After that, we fitted a set of generalized additive models (GAM) [37] that explored the effects of the disease management strategies on TB control. In this case, TB infection status (the categorical dependent variable with two modalities: 1 if TBLL were present or a positive culture obtained, and 0 otherwise) was analyzed taking into account as dependent variables the harvesting season, the number of harvested wild boar, age of animals (only juveniles, yearling and adults were retained for this analysis due to the chronic character of bovine tuberculosis and because few TB infected piglets were captured during the study period), cattle removal (a categorical variable with two modalities: pre-cattle removal and post-cattle removal) and their two-way interactions. The variable harvesting season was included in all models given that we aimed to explore temporal trends in TB infection rate.

Since the effects of harvesting pressure on TB occurrence would not be immediate, we considered several harvesting pressure-related variables by accounting for the accumulated number of wild boar harvested in one, two, and three harvesting seasons previous to the current one. It was impossible to consider beyond the three previous seasons because these data were not available for harvesting season 4. Nevertheless, owing to the correlation between these variables (e.g., R^2^ = 87%, t = 6.3, p>0.001 for the correlation between the accumulated number of wild boar harvested three and two seasons before the current one), only two explanatory harvesting pressure-related variables were retained for the analysis: the number of wild boars harvested in the previous harvesting season (Harv1) and the accumulated number of wild boar harvested in the three previous seasons (Harv3). Following the same rationale, no model simultaneously included harvesting season and the Harv3 as explanatory variables due to high correlation (R^2^ = 79%, t = 4.83, p>0.001). Finally, we explored whether the observed TB trends in TBA differed from those in OA and DA by a model (GAM) including the interaction between harvesting season and zone (TBA, OA and DA).

Finally, we explored the potential mechanisms through which increased wild boar harvesting influenced population structure and hence TB dynamics. For this purpose, we fitted a set of linear models (LM) to explore whether total wild boar abundance or the percentage of juveniles were influenced by Harv1 or Harv3. Closed scrublands on steep slopes predominate in our region, which hinders census through direct observation of animals as well as application of indirect methods based on faecal droppings for abundance estimates [12]. For this reason, the number of sighted wild boar (including those hunted) divided by the number of participating hunters in the hunting journey was considered a proxy for wild boar abundance (see [38] for a revision on census methods for wild boar).

In all cases, we followed a model selection procedure based on the information-theoretic approach and the Akaike's Information Criterion [39]. Subsequently, we estimated the Akaike weight (*w*i,), defined as the relative probability that a given model is the best model among those being compared. Once the best model was selected we confirmed the general assumptions of GAM and LM following the previously published recommendations [37], [40]. Statistical analyses were performed using “mgcv” package version 1.7-12 [37] of the statistical software R version 3.0.2 [41].

## Results

### Prevalence of TB and genotype mapping

Twenty-four percent of wild boars showed TBLL (179/745). Sixteen percent had a positive culture of *Mycobacterium* sp. (121/745) and, based on both TBLL and/or a MTBC positive culture, the overall prevalence of TB in the NGRPTB was 24.7% (21.6–27.9 at 95% CI; n = 184). Distribution of isolates into mycobacterium species and spoligotypes is presented in Table 1. Tuberculous infection by members of the MTBC was confirmed in 103 wild boars (97 *M. bovis* y 6 *M. caprae* isolates). The remaining eighteen isolates corresponded to species of the genus *Mycobacterium* other than MTBC. Moreover, eighteen wild boars without TBLL displayed positive mycobacteria cultures (four to *M. bovis*, one to *M. caprae* and 13 to non-tuberculous mycobacteria), and no mycobacteria were recovered from 76 wild boars with TBLL. Two wild boars detected in DA with miliary TBLL in the lung parenchyma were ultimately diagnosed as pulmonary botryomycosis due to infection with *Staphylococcus aureus*. None of the ibexes harvested had TBLL or a positive *Mycobacterium* sp. culture.

**Table 1:** Distribution of 121 isolates into mycobacterium species and spoligotypes in eight harvesting seasons.

Harvesting season	*Mycobacterium bovis*									*Mycobacterium caprae*	Non-MTBC mycobacteria
*4			SB0121 (2)				SB1095 (2)	SB1192 (1)			
*5	SB0119 (1)	SB0120 (1)	**SB0121** (7)				SB1095 (1)	SB1192 (2)	SB1336 (6)	SB0415 (1)	NA (2)
*6			SB0121 (4)			SB0295 (2)	SB1095 (4)	SB1192 (1)	SB1336 (4)		NA (3)
*7			**SB0121** (15)			SB0295 (2)	**SB1095** (5)	**SB1192** (1)		SB0415 (3)	NA (6)
8			SB0121 (2)	SB0140 (1)	SB0294 (1)	SB0295 (1)	SB1095 (7)			SB0415 (1)	NA (2)
9			SB0121 (1)		SB0294 (3)		SB1095 (1)			SB0415 (1)	
10			SB0121 (2)		SB0294 (1)		SB1095 (3)				NA (1)
11			SB0121 (3)	SB0140 (1)			SB1095 (8)	SB1192 (2)			NA (4)
TOTAL	1	1	36	2	5	5	31	6	10	6	18

*Mycobacterium tuberculosis* complex spoligotypes and number of isolates (in brackets) in 121 harvested wild boars in eight harvesting seasons starting in the period 2004-05 (4^th^) and ending in 2011–12 (11^th^). Asterisks indicate those harvesting seasons in which TB-infected cattle were present in TBA. In bold, TB spoligotypes also isolated from cattle in the respective season.

Ten different spoligotypes have been identified from the 103 MTBC-infected wild boar and the most frequent profiles were SB0121 (n = 36) and SB1195 (n = 31), detected in the reserve from 2004 to 2012. The remaining eight spoligotypes were sporadically isolated in wild boar (n = 36). In the five isolates from cattle, four spoligotyes were identified (SB0121, SB1095, SB1192 and SB1685) and three of them (SB0121, SB1095, and SB1192) were also shared with wild boar. Five of the spoligotypes detected in TBA (SB0121, SB0294, SB0415, SB1095 and SB1192) have also been isolated from wild boar harvested in OA, whereas only the *Mycobacterium caprae* (SB0415) has been detected in DA. Ninety-nine out of 103 MTBC isolates (no DNA was available for four isolates) were also characterized by MIRU-VNTR typing with 9 loci, and 13 MIRU-VNTR types were obtained when ETR-A, ETR-B, QUB11a and QUB322 loci were analysed, with the most frequent being MIRU-VNTR (MV) type MV0006 (n = 59)(see Table S1). In general, the five remaining VNTR markers (QU11b, MIRU4, MIRU31, MIRU26 and QUB26) did not provide additional information and the isolates were clustered using only the four most polymorphic loci (see Table S2).

Attending to age classes, the prevalence of TB was 58.6% amongst adults, 32.2% in juveniles and yearlings and 6.3% in piglets. The occurrence of TBLL in different anatomical regions (localized versus more extended or generalized) are presented in Table 2. Most tuberculous wild boar had lesions in the head lymph nodes (109/115, 95%), while only 14/115 and 6/115 had lesions only in intrathoracic (mediastinic or bronchial) or mesenteric lymph nodes, respectively.

**Table 2:** Occurrence of tuberculosis-like lesions in different anatomical regions of wild boar.

TB LESION DISTRIBUTION PATTERNS (n = 115)	
**One anatomical region**	89%(102)
** Head only**	83.5%(96)
** Thorax only**	4.3%(5)
** Abdomen only**	0.8%(1)
**Two or more anatomical region**	11%(13)
** Head an thorax**	7%(8)
** Head and abdomen**	3.5%(4)
** Thorax and abdomen**	0
** Head, thorax and abdomen**	0.9%(1)
**Submandibular lymph nodes**	92%(106)
**Retropharyngeal lymph nodes**	16.5%(19)
**Bronquial or mediastinic lymph nodes**	12%(14)
**Lungs parenchyma**	1.7%(2)
**Mesenteric lymph nodes**	5%(6)
**Abdominal viscera**	0

Percentage of wild boars showing localized of generalized specific TBLL in different anatomical regions amongst 115 individuals in which complete post-mortem examination was performed.

### Spatial pattern of TB in the game reserve

**Figure 2:**
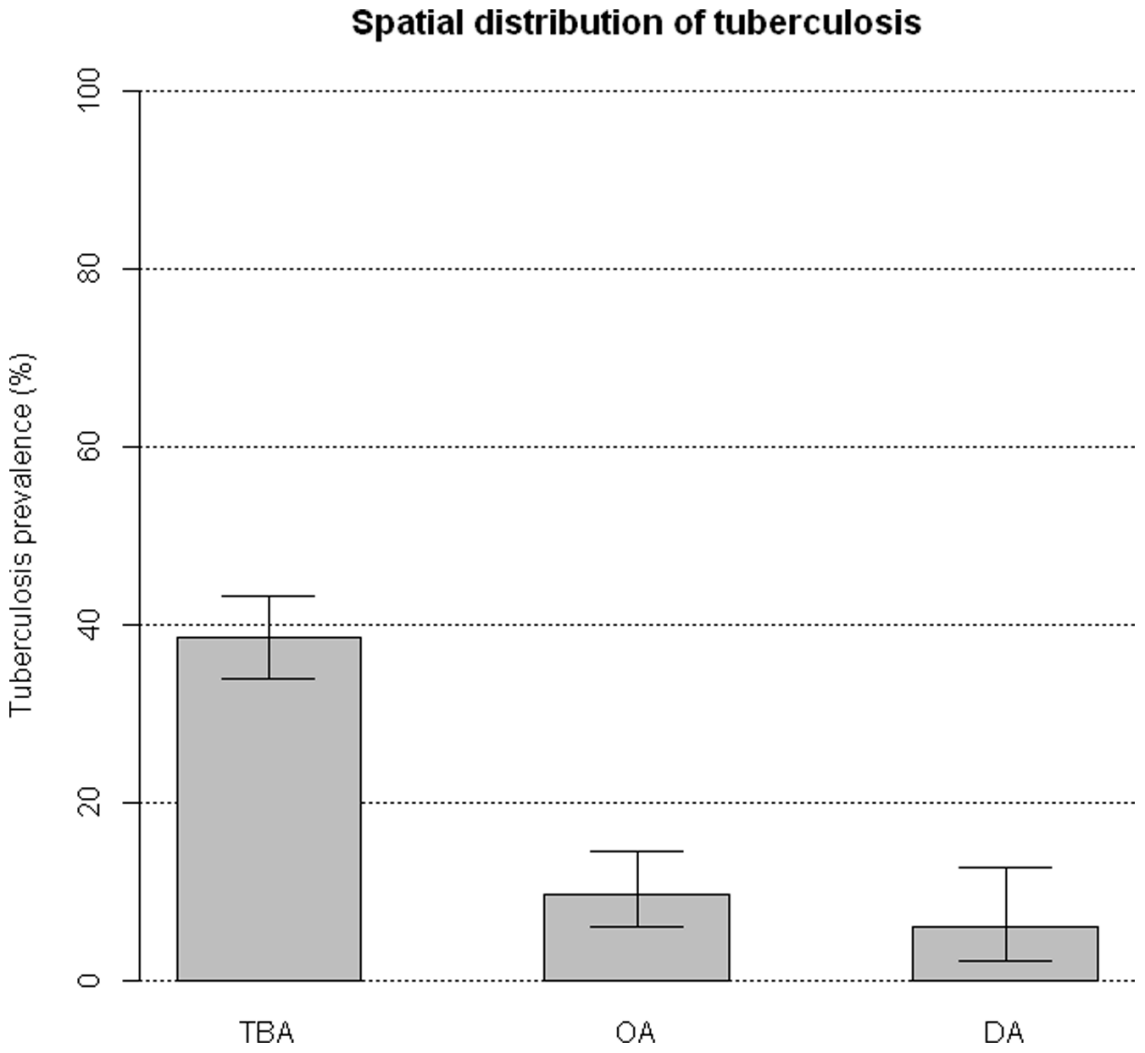
Spatial pattern of tuberculosis in the National Game Reserve “Ports de Tortosa i Beseit”. Prevalence of tuberculosis in wild boars harvested in the National Game Reserve “Ports de Tortosa i Beseit” in three defined areas: the main tuberculosis area (TBA), outlying areas to TBA (OA) and distant areas to TBA (DA1 and DA2 were pooled). Confidence interval at 95% is represented.

85.3% of the TB positive boars (168/197) were harvested in TBA, which accounts for an overall prevalence of 38.5% in this zone (33.9–43.2 95% CI, 168/436 examined wild boar) and results between 4 and 6.42 times higher than in OA or DA, respectively (Fisher test  = 85.62, d.f. = 2, p-value <0.001, Figure 2).

### Effects of disease management on TB control

**Figure 3:**
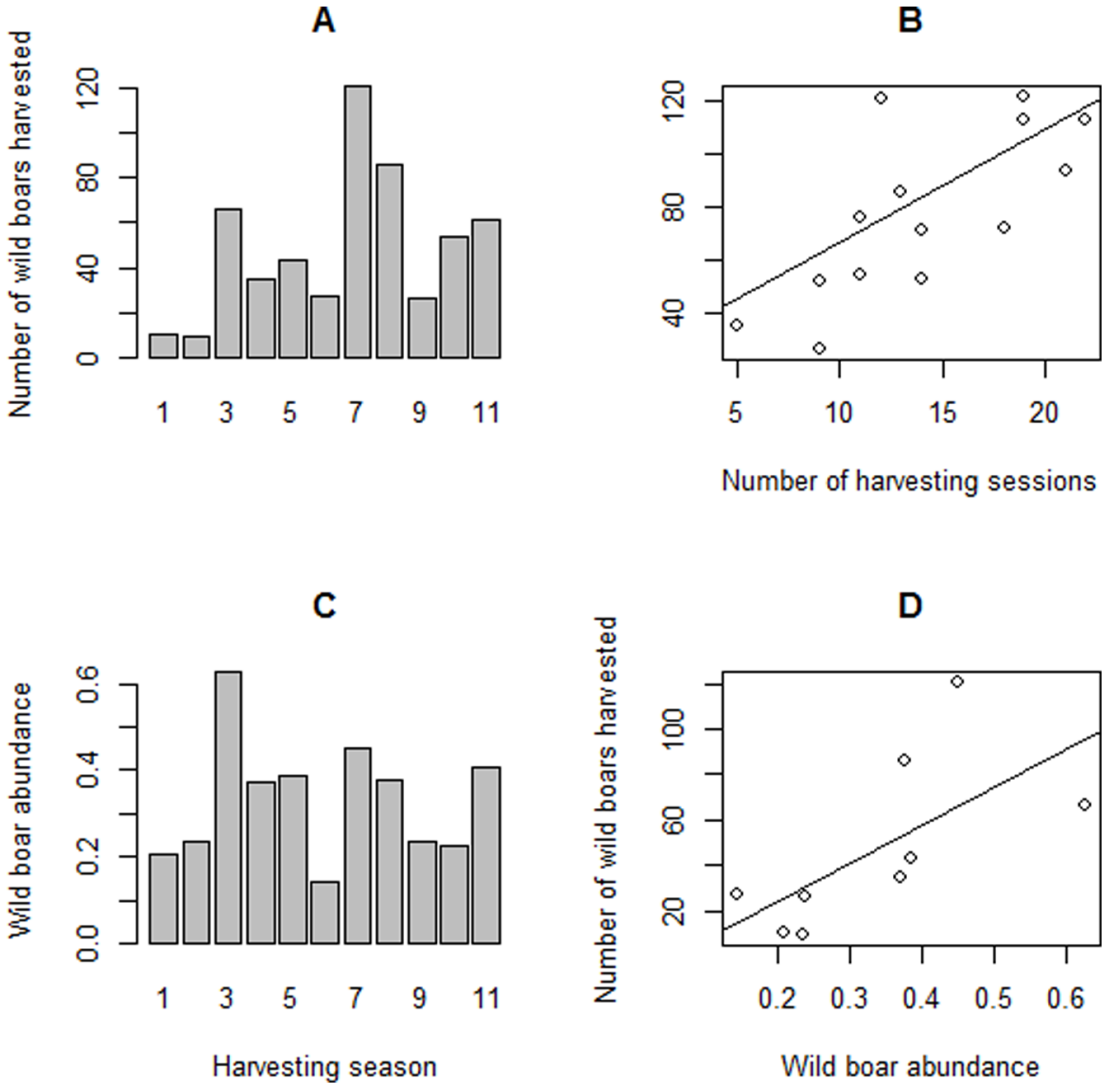
Relationship amongst hunting pressure, wild boar abundance and the number of wild boar harvested. Number of harvested wild boars (a) and abundance (b) per game season; Graphical representation of positive correlation between the number of harvesting sessions (c) and abundance (d) and the number of harvested wild boars.

Harvesting pressure nearly tripled (e.g., increased 2.6 times); in fact, only 3.7 beats per game season occurred previous to TB detection (period 2001–2004), whereas 9.6 harvesting actions per game season (including both hunting and box-trapping) occurred after TB detection in wild boar. In general, the increase in the number of harvesting sessions resulted in a higher number of harvested boars during the whole study period (β = 4.3, SE = 0.22, t-value  = 19.36, p<0.001, R^2^ = 36.9%, Figures 3a and 3b). But the number of harvested boars in every harvesting period was also positively correlated to their abundance in the corresponding period (β = 218.14, SE = 9.26, t-value = 23.55, p<0.001, R^2^ = 60.7%, Figures 3c and 3d). Hence, the peak in the number of harvested wild boar in the game season 3 would be more related to the high abundance in this season whereas the peak observed in harvesting seasons 7 and 8 would be more related to harvesting effort.

**Figure 4:**
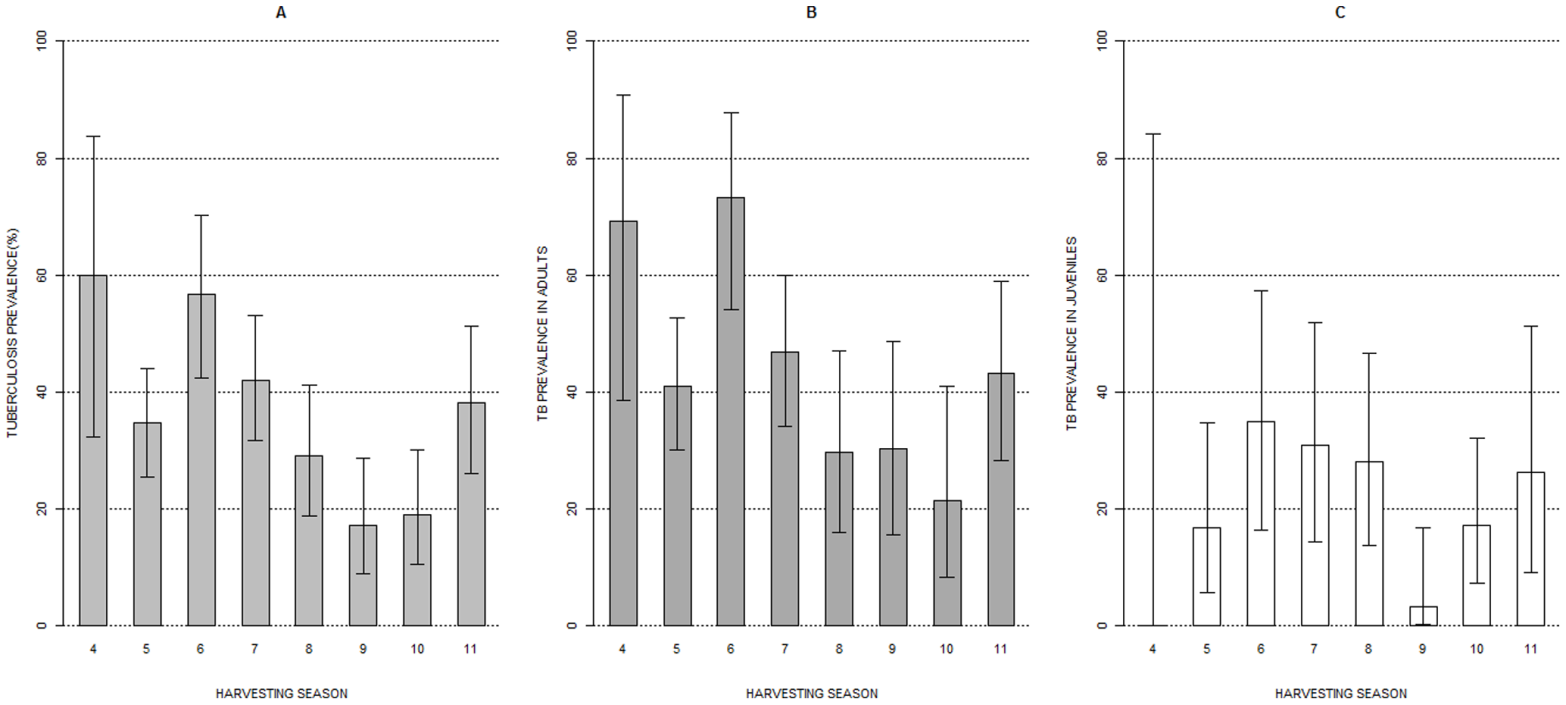
Temporal evolution of tuberculosis prevalence in wild boar. Overall prevalence of tuberculosis (a), prevalence in adults (b) and in juveniles (c) per harvesting seasons in TBA from 2004 to 2011. Cattle removal occurred in May 2008 (this is by the end of harvesting season 7).

On the other hand, the prevalence of TB infection displayed a temporary pattern in TBA (Figure 4). According to our model selection procedure, the cattle removal, the accumulated number of wild boar harvested in the last three harvesting seasons (Harv3) and the age of wild boar were the main factors for explaining the probability of TB in the wild boar (*W_i_*_Harv3 * Cattle removal + Age_ = 1; that explained 15% of the observed variability in the probability of TB infection; Table 3). The second competing model, which included Harv3 and age, was at 31.23 units from the best model, and thus a candidate with little support for explaining the observed patterns [39]. On the other hand, both juveniles and adults followed the same temporal pattern (the model including the interaction with age was at 32.13 units from the best model), but as expected, the probability of TB infection for the young animals was half that for the adults (β _Yearlings_ = −1.0023, SE = 0.2231, Z = −4.492, p-value <0.001). Concerning the effect of TB management, neither cattle removal nor wild boar harvesting were able to shape temporal TB dynamics independently (models including the single effects of these management strategies were more than 54 units from the best model).

**Table 3:** Models considered for explaining the probability of tuberculosis infection in the wild boar.

Biological models	K	AIC	Δ_i_	*w_i_*
Harv3 * Cattle removal + Age	8	590.09	0.00	1
Harv3 + Age	5	621.32	31.23	0
Harv3 * Age + Cattle removal * Age	8	622.22	32.13	0
Harv3 + Age + Cattle removal	6	622.94	32.85	0
Harv3 * Age	6	636.89	46.80	0
Game season + Age + Cattle removal * Harv1	8	641.6	51.51	0
Game season + Age	5	642.7	52.61	0
Game season + Age + Cattle removal + Harv1	7	643.07	52.98	0
Game season + Age + Harv1	6	643.17	53.08	0
Game season * Harv1 + Age	6	644.03	53.94	0
Game season + Age + Cattle removal	6	644.54	54.45	0
Game season + Age * Harv1	7	644.85	54.76	0
Game season + Age * Cattle removal	7	645.59	55.50	0
Game season + Cattle removal * Age + Harv1* Age	9	646.16	56.07	0
Mo	1	774.22	184.13	0

Model selection based on generalized additive modelling for exploring the temporal variation in the probability of tuberculosis infection determined in 267 adult, 97 juvenile and 70 yearling wild boars harvested in the main tuberculosis area and outlying areas (TBA and OA) in the National Game Reserve “Ports de Tortosa i Beseït”. Harv1 means the total number of wild boars harvested in the previous harvesting season, whereas Harv3 is the total number of wild boars harvested during the three seasons before the current one. K  =  effective number of parameters in the additive modelling, AIC  = Akaike Information Criterion, Δ*_i_*  =  difference of AIC with respect to the best model, *w_i_*  =  Akaike weight, Mo  =  null model only with the constant term. In bold the best model for explaining the observed TB probability of infection.

**Figure 5:**
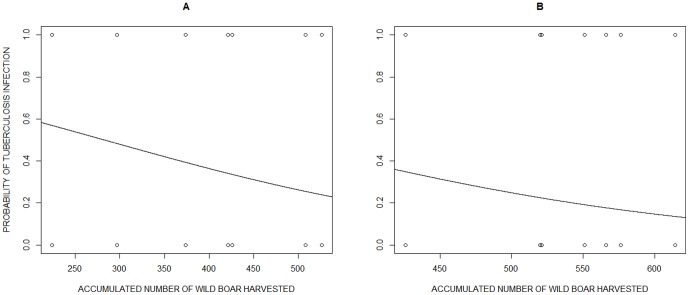
Effect of wild boar harvesting on the probability of tuberculosis infection. Effect of wild boar harvesting before (a) and after (b) cattle removal on the probability of TB infection in wild boars harvested in the main tuberculosis area (TBA) in the National Game Reserve “Ports de Tortosa i Beseit”, Catalonia, north-eastern Spain.

In general (all age classes included), the TB prevalence before cattle removal was 49% (42–56 at 95% CI; n = 221) and decreased to 28% (22–34 CI 95%; n = 215) after cattle removal, with an odds ratio of 2.46. Such odds ratio increases to 3.3 when considering only individuals aged over 1 year (69% TB prevalence before vs 40% after cattle removal).However, only the interaction of the two measures resulted in the decrease in the probability of TB infection. Furthermore, intensive wild boar harvesting was more effective in reducing TB infection rates before (deviance explained  = 16 %) than after cattle removal (deviance explained  = 9%), perhaps because the TB prevalence was 1.9 times lower after cattle removal (e.g., for an accumulated number of wild boar harvested of 500, the probability of TB infection was 1.2 times greater before cattle removal; Figure 5). Finally, TB dynamics differed between TBA, where the management actions were carried out, and OA or DA, as shown by the significant interaction between harvesting season and the study area (Chi-square  = 12.38, p-value 0 0.005, 28.5% deviance explained).

### Effects of intensive harvesting on the wild boar population structure

**Figure 6:**
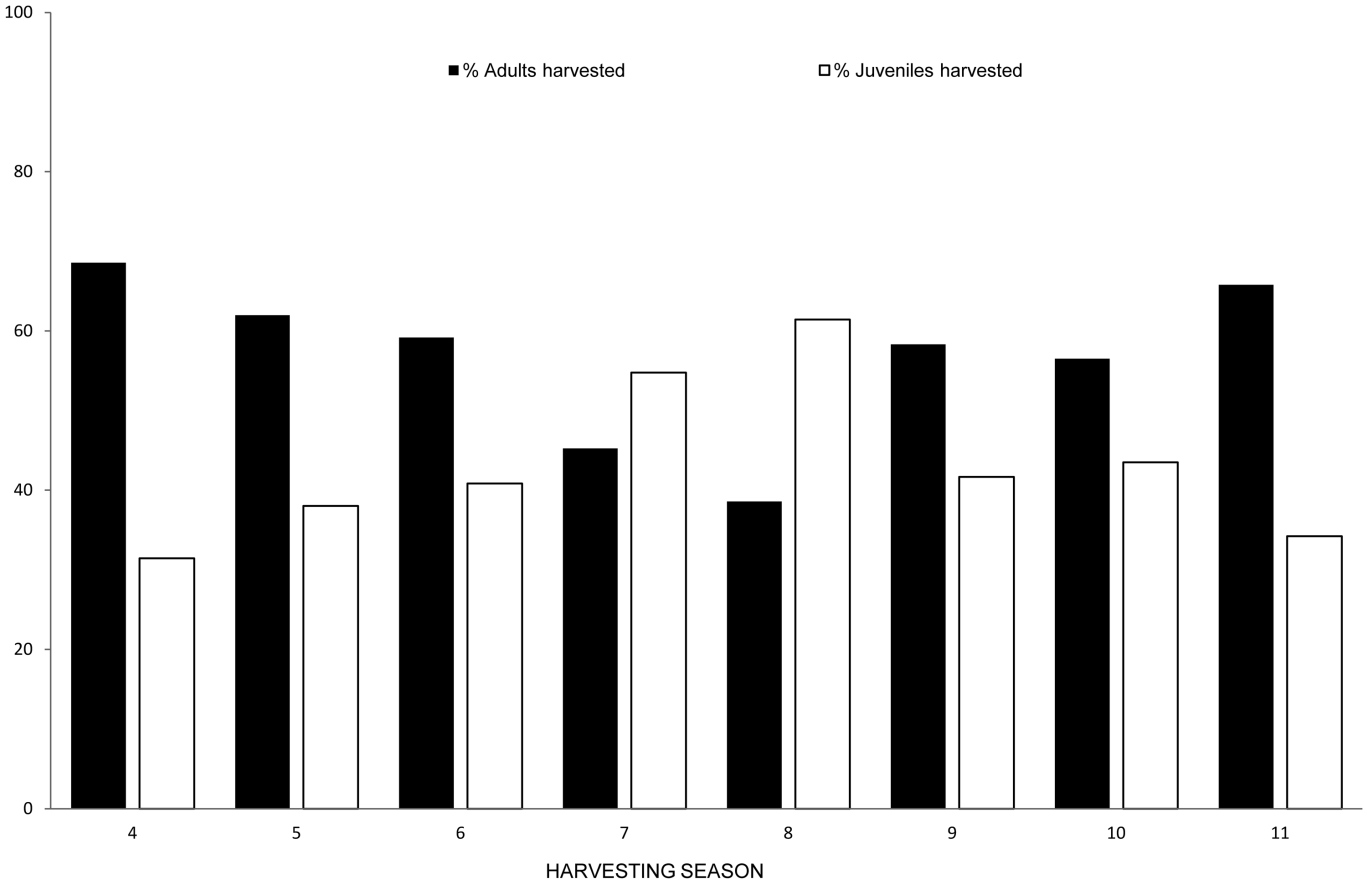
Temporal evolution of wild boar population age structure. Percentage of adult (a) and young (juveniles and yearlings) b) wild boars harvested in TBA per harvesting season.

The intense harvesting of wild boar in TBA had an effect on that wild boar population. The age structure of the TBA wild boar population varied over the study period due to an increment of young (juveniles + yearlings) animals and a decrease of adult boars (Figure 6). This was not reflected in OA or DA. Actually, this trend could be a consequence of TB management because of the relationship between the harvesting pressure in the previous seasons and the increment of young boars harvested in the TBA (β _Harv1_ = 0.05, SE = 0.02, Z = 2, p-value <0.05, R^2^ = 35.2%). On the other hand, the increment of harvesting pressure did not influence wild boar population abundance (β _Harv1_ = −0.0003, SE = 0.0003, Z = −0.9, p-value  = 0.4).

## Discussion

Whereas southern Spain is a hotspot of tuberculosis in wildlife, reports of the disease are scarce in the northern part of the country, where conditions of wildlife are more similar to those in the rest of Europe (no fencing, no artificial feeding and lower densities). This study provides evidence that high TB prevalence is also possible in free-ranging and non-intensively managed (i.e., not overcrowded, non-fenced and without supplemental feeding) wild boar populations living in the absence of deer (fallow deer and red deer). In fact, deer are present in most of the tuberculosis scenarios in which wild boar is implicated [13], [42], including large natural protected areas (e.g., Doñana National Park (DNP)) [43]. Instead, the Iberian ibex, a wild caprinae, is present in the scenario considered here. Previous studies have suggested that this wild caprinae does not play a significant role in the epidemiology of TB [44], and the presented results increase the previous sample size reinforcing this idea. The TB prevalence (TBLL and/or culture positive) found in our study area is amongst the highest described in wildlife, reaching values around 70% in adults in some harvesting seasons (see Fig. 5). Similar values have been observed in overcrowded and intensively managed wild boar populations in the central Iberian Peninsula [9], and higher, up to 92% in free-ranging wild boars from DNP [43] and 100% in feral pigs from New Zealand [6]. The post-mortem examination is an easy-to-perform and inexpensive first option for the presumptive diagnosis of TB in game species. However, prevalence of TB in our study may have been slightly underestimated since histopathology was not routinely performed and a low percentage of infected wild boar with microscopic lesions and a false-negative bacteriological culture result could have gone unnoticed. Actually, a negative culture status is not a guarantee that a wild boar is not infected [34], as reinforced in our study by animals with TBLL and a negative culture (76/179) or vice versa, positive culture without TBLL (18/566). Overestimation of TB prevalence based on TBLL has been suggested due to infection by other pathogens [9]. The *Staphylococcus aureus* causing pulmonary botryomycosis was detected in DA, far from TBA, whereas infection by MTBC was confirmed in a significant percentage (90/157) of the wild boar with TBLL in TBA, and thus overestimation is improbable.

Generalized lesions in wild boar from the NGRPTB were rare (11%) as compared to populations from south central Spain, where values around 60% have been repeatedly observed and attributed to the early infection of young animals favoured by unnaturally high densities and spatial aggregation [9], [45]. In fact, disease progression is likely to be more severe in immature individuals because of reduced immunocompetence [45], [46]. The importance of the route of infection on the TB lesion pattern is still unclear. De Lisle, for example, associated the localized lesions in the head lymph nodes of feral pigs from New Zealand to infection through scavenging of tuberculous carrion [46], whereas Martin-Hernando and cols. attributed both localized and generalized patterns to either respiratory (air-borne infection by frequent direct oronasal contact behaviour between wild boar) or digestive (food and water) infection routes [45]. Based in the literature and our own data, we could hypothesize that lesion distribution may be positively correlated to exposure, as it would increase the probability of a wild boar getting infected in early life and by several routes. But other factors may also determine lesion pattern. Garcia-Jimenez and cols., for example, described a higher load of mycobacteria in lesions caused by *M. caprae* (scarce in our study), which could result in higher excretion rates and, subsequently, exposure for other individuals in the population [47]. Additionally, genetic factors have been related to the ability of both wild boar to limit infection (host resistance) and *Mycobacterium bovis* to circumvent host immune responses and establish infection (pathogen mechanisms of virulence) [48]. Furthermore, climate and food availability would also be key factors driving disease severity. In fact, the problem of tuberculosis in wild boar seems to be concentrated in south-west Spain [9], where long dry summers [49] and the homogeneous habitat and high densities of ungulates [24] often result in food and water shortages for nearly half the year. Our study is located in the northern third of the Iberian bio-region 5 [24], corresponding to coastal areas where seasonal food shortages are not likely to lessen the ability of wild boar to cope with TB infection. The landscape composition may also play a role, as the heterogeneous habitat of our study area could revert to greater availability and variety of food and water resources, thus minimising shortage periods and improving micronutrient intake. Altogether, these factors may determine fitness, immunocompetence and even coinfection-related variations in susceptibility to infections by regulation of the pathogen community and pathogen load of animals [50], [51]. However, all of these arguments are to date speculative, and hence further research is necessary to explain the lack of generalized lesions in our study area.

To our knowledge this study reveals a local MTBC spoligotype diversity never before described in the scientific literature [10], [52], [53]. The strains shared with sympatric livestock point to cattle as the original source of infection for wild boar in the TBA. This diversity of spoligotypes may suggest repeated introductions of infected cattle in TBA in the past, which favoured the spread of new spoligotypes to the wild boar population. On the other hand, the genotyping analysis grouped several spoligotypes within the same MIRU-VNTR type. The MV0006 type (n = 59) included the majority of the isolates with the spoligotypes SB0121, SB1095, and SB1685 (only found in cattle), and the loss of spacers within the DR locus could also suggest the evolution and fitness of the new strains in this area, as previously described in other regions [52]. The fitness of the SB0121 and SB1095 strains in wild boar became clear when TB increased at seasons 10 and 11, and these profiles were maintained in the area whereas other genotypes disappeared. The SB0121 is the most prevalent spoligotype in the Iberian Peninsula in cattle, goats and wildlife [53], [54] and also the most frequent profile in our study area. By contrast, the second predominant spoligotype in our study area, SB1095, may be biased toward northern Spain [54]. On the other hand, the appearance of only one spoligotype of *M. caprae*, SB0415, and in distant areas probably indicates that past infections remain from infected flocks of domestic goats, very common a few decades ago. Currently, the presence of caprine livestock in the NGRPTB is anecdotic.

The marked spatial pattern of TB in the present study is, at least, unexpected given the absence of physical barriers in the study area other than orography. This concentration of tuberculosis reinforces the hypothesis of cattle as a source of infection for wild boar in TBA. The intensive culling of badgers has been observed to produce unexpected effects such as TB dispersal or increased prevalence both in cattle and badger populations in the UK [16] due to increased movements of surviving individuals [55]. In our study, the spatial concentration of wild boar TB in our study was maintained throughout the study period despite culling. This is probably due to their preference to staying in TBA, where water springs and food (fruit trees and farming by-products) are available year-round. Moreover, wild boar leaving the TBA can be hunted in OA or more distant areas since wild boar is hunter-harvested everywhere in the surrounding areas.

As mentioned, the efficacy of the culling method for disease management in wildlife is subject to some controversy [16], [19], also in the specific case of the European wild boar [17], [18]. In our study, a temporary reduction of TB prevalence in wild boar as a result of both increasing harvesting and removing cattle was achieved between harvesting seasons 7 and 10. For comparison, the prevalence of TB in feral swine decreased from 20% in 1980 to 3.2% in 1983 in the Hawaiian island of Molokai, after an infected cattle herd was removed and intense hunting pressure reduced feral swine density [56]. Our model selection supported the same temporally decreasing pattern in TB occurrence for both juveniles and adults, which could be due to the reduction of force of infection in juveniles, to the incorporation of young TB-free animals and the reduction of infected adults, respectively. However, it was impossible to disentangle the effects of wild boar culling and cattle eradication on TB reduction. Nonetheless, the role of cattle as a source of TB infection appears clear as the prevalence in wild boar fell to half after livestock removal. On the other hand, intensive culling did not result in a reduction of wild boar abundance, maybe due to the dispersion of young animals from neighbouring areas or to increased turnover derived from compensatory reproduction observed in intensively-hunted wild boar populations [57], [58]. Hence, the use of culling as a measure for reducing density of infected animals and consequently opportunities for disease transmission did not seem to work in our study system. Thus, the rejuvenation of wild boar populations and the reduction of force of infection would be the most plausible mechanism for explaining the reduction observed in TB prevalence. This highlights the need for integrated holistic control strategies.

It is important to highlight that the wild boar harvesting was more effective before cattle removal, indicating that intensive culling is an effective first intervention measure, especially in areas with high TB prevalence [17]. It is also interesting to observe that the disease still remains in our wild boar population several years after eradication in cattle. This fact lends clear support to the role of wild boar as a true reservoir of TB in the Mediterranean context [8], even at low densities if other risk factors take place. Apart from that, the re-introduced herd in TBA remains negative based on the periodical skin tests performed to date, probably due to the improvement of farming practices. Despite the efforts made, TB increased in wild boar at the end of the study period (e.g., harvesting period 11 or 2011-2012), which would confirm the lack of effectiveness of intensive harvesting for TB control when prevalence is low. This could be attributed to the collateral increase of juveniles and to the decrease in the harvesting efforts during the last years after peaking in periods 7 and 8. This supports that disease control measures must be continued over time and intensively enough to achieve the required efficacy and to counteract the temporary nature of beneficial effects. Intensive culling has achieved TB eradication in the wild only through massive and sustained programs [59]. This is crucial if we bear in mind that we are dealing with an abundant species such as wild boar that locally reaches pest levels and whose ecological elasticity and behaviour allows them to cope with high harvesting rates [57], [58]. In fact, some authors propose more selective harvesting strategies to improve efficacy for achieving population reduction [57]. For example, Bieber and Ruf propose that reducing juvenile survival will have the largest effect on population growth rate under good environmental conditions, whereas strong hunting pressure on adult females will lead to the most effective population control in years with poor conditions [60]. Box-trapping was quite selective for young animals in our study (70% of box-trapped wild boar in our study were below 2 years old), hence this could be a good methodological option for the first assumption.

### Concluding remarks

Active disease surveillance in wildlife makes clear its value and discloses the first high prevalence focus of tuberculosis described in wildlife in the northern half of the Iberian Peninsula. Until now, this focus went unnoticed despite affecting a high percentage of the local wild boar population. Moreover, although TB in wildlife was not assumed to be a cause of concern in these latitudes, the conditions necessary for the onset of this focus have occurred within a protected area where risk factors such as fencing, feeding, overcrowding and/or varied host communities are lacking. Hence, the potential risk for tuberculosis emergence in wildlife populations under certain conditions should not be neglected in the future. Both our evidence and that found in the literature point to exposure as a key factor to understanding lesion patterns of affected individuals. Nevertheless, a better understanding of this question as well as of other factors determining susceptibility and virulence could derive implications for management aimed at reducing generalized patterns and, consequently, curbing infected individuals and exposure of healthy susceptible ones. Factors determining the movements of wild boar may also be of interest to TB management in unfenced areas.

Active disease surveillance in wildlife makes clear its value and discloses the first high prevalence focus of tuberculosis described in wildlife in the northern half of the Iberian Peninsula. Until 2004, this focus went unnoticed despite affecting a high percentage of the local wild boar population. A TB in wildlife was not anticipated to be of concern in these latitudes since the assumed conditions necessary for the onset of the disease such as fencing, feeding, overcrowding and/or varied host communities were lacking. Hence, the potential risk for tuberculosis emergence in wildlife populations under more natural conditions should not be neglected in the future. Both, our evidence and documented cases in the literature point towards early and intense exposure as a key factor to understand lesion patterns of affected individuals. Nevertheless, a better understanding of this question as well as of other factors that drive susceptibility and virulence, it will help to design management practices to reduce generalized patterns and, consequently, reduce exposure of healthy susceptible ones. Factors determining the movements of wild boar may also be of interest to TB management in unfenced areas.

Some evidence suggests that poor farming practices may have occurred in this area in the past. This should be noted especially by the managers of natural areas where interaction between livestock and wildlife may occur, in order to apply preventive measures. Once again, but for the first time in TB, we have shown that it is possible to address the control of a multi-host pathogen in a wild host population via the management of the domestic counterpart [61]. However, the need for holistic control strategies is highlighted, as reduction but not eradication was achieved with the applied measures. Culling is probably the least expensive measure that can be applied, which makes it valuable in the current scenario of economic shortages in the EU (affecting countries in the Mediterranean basin more severely, where this problem [TB in wild boar populations] occurs). Our “culling” experience in TB-infected wild boar adds to the recent ones [17], [18] with clarifying intermediate results. As observed by Boadella and cols., culling seems to be an effective first intervention measure to be applied in high prevalence foci, whereas efficacy tends to decrease as prevalence does [17]. However, estimating the threshold required for a significant reduction under natural conditions can be difficult. Hence, to achieve either eradication or a significant decrease of prevalence, culling must be continued over time and intensively enough to achieve the required efficacy and to counteract the temporary nature of beneficial effects. Alternatively, the applicability of more selective harvesting strategies to improve efficacy for achieving population reduction are another area to explore.

## Supporting Information

Table S1 **MIRU-VNTR results and analysis of the** ***M. bovis*** **and** ***M. caprae*** **isolated from wild boar.** ^a^ MIRU-VNTR loci with corresponding alias. ^b^ MIRU-VNTR types obtained with the four-loci approach including ETR-A, ETR-B, QUB11a and QUB3232. Table is arranged according to the MVtype in ascending order. ^c^ Eight out of 99 isolates are not included in the table due to failure in one or more loci or multiple bands in some loci. Grey indicates the MIRU-VNTR profile of the five cattle isolates. The bovine isolate with the SB1685 profile (not included in the table because was only present in cattle) showed the most frequent MV0006 type and the SB0121 spoligotype.
(XLSX)

Table S2 **Genotyping analysis of the** ***M. bovis*** **and** ***M. caprae*** **isolated from wild boar.** Genotyping analysis (combination of spoligotypes and MIRU-VNTR types) of the *M. bovis* and *M. caprae* isolated from wild boar. ^a^ Eight out of 99 isolates genotyped by MIRU-VNTR are not included in the table due to failure in one or more loci or multiple bands in some loci. ^b^ MIRU-VNTR types obtained with the four-loci approach including ETR-A, ETR-B, QUB11a and QUB3232. Grey indicates the season and genotype of the cattle isolates. The bovine isolate with the SB1685 profile belongs to season 7 but is not included in the table since no wild boar were detected with this spoligotype.
(XLSX)
